# Proteomic identification of membrane-associated placental protein 4 (MP4) as perlecan and characterization of its placental expression in normal and pathologic pregnancies

**DOI:** 10.7717/peerj.6982

**Published:** 2019-06-20

**Authors:** Nikolett Lilla Szenasi, Eszter Toth, Andrea Balogh, Kata Juhasz, Katalin Karaszi, Oliver Ozohanics, Zsolt Gelencser, Peter Kiraly, Beata Hargitai, Laszlo Drahos, Petronella Hupuczi, Ilona Kovalszky, Zoltan Papp, Nandor Gabor Than

**Affiliations:** 1 Systems Biology of Reproduction Research Group, Institute of Enzymology, Research Centre for Natural Sciences, Hungarian Academy of Sciences, Budapest, Hungary; 2 MS Proteomics Research Group, Institute of Organic Chemistry, Research Centre for Natural Sciences, Hungarian Academy of Sciences, Budapest, Hungary; 3 First Department of Pathology and Experimental Cancer Research, Semmelweis University, Budapest, Hungary; 4 Department of Medical Biochemistry, Faculty of Medicine, Semmelweis University, Budapest, Hungary; 5 West Midlands Perinatal Pathology, Birmingham Women’s Hospital, Birmingham, UK; 6 Maternity Private Clinic of Obstetrics and Gynecology, Budapest, Hungary; 7 Department of Obstetrics and Gynecology, Semmelweis University, Budapest, Hungary

**Keywords:** Miscarriage, Placenta, Preeclampsia, Proteoglycan, Pregnancy

## Abstract

**Background:**

More than 50 human placental proteins were isolated and physico-chemically characterized in the 70–80s by Hans Bohn and co-workers. Many of these proteins turned to have important role in placental functions and diagnostic significance in pregnancy complications. Among these proteins was membrane-associated placental protein 4 (MP4), for which identity or function has not been identified yet. Our aim was to analyze the sequence and placental expression of this protein in normal and complicated pregnancies including miscarriage, preeclampsia and HELLP syndrome.

**Methods:**

Lyophilized MP4 protein and frozen healthy placental tissue were analyzed using HPLC-MS/MS. Placental tissue samples were obtained from women with elective termination of pregnancy (first trimester controls, *n* = 31), early pregnancy loss (EPL) (*n* = 13), early preeclampsia without HELLP syndrome (*n* = 7) and with HELLP syndrome (*n* = 8), late preeclampsia (*n* = 8), third trimester early controls (*n* = 5) and third trimester late controls (*n* = 9). Tissue microarrays were constructed from paraffin-embedded placentas (*n* = 81). Slides were immunostained with monoclonal perlecan antibody and evaluated using light microscopy and virtual microscopy. Perlecan was also analyzed for its expression in placentas from normal pregnancies using microarray data.

**Results:**

Mass spectrometry-based proteomics of MP4 resulted in the identification of basement membrane-specific heparan sulfate proteoglycan core protein also known as perlecan. Immunohistochemistry showed cytoplasmic perlecan localization in syncytiotrophoblast and cytotrophoblasts of the villi. Perlecan immunoscore decreased with gestational age in the placenta. Perlecan immunoscores were higher in EPL compared to controls. Perlecan immunoscores were higher in early preeclampsia without and with HELLP syndrome and lower in late preeclampsia than in respective controls. Among patients with preeclampsia, placental perlecan expression positively correlated with maternal vascular malperfusion and negatively correlated with placental weight.

**Conclusion:**

Our findings suggest that an increased placental perlecan expression may be associated with hypoxic ischaemic injury of the placenta in miscarriages and in early preeclampsia with or without HELLP syndrome.

## Introduction

A total of 15% of clinically recognized pregnancies end with miscarriage (Prager, Dalton & Allen, 2015), and ∼25% of the remainder are affected by the great obstetrical syndromes (e.g., preeclampsia, intrauterine growth restriction). Pregnancies impacted by these syndromes are complex, since mothers, fetuses or both can be severely affected by their high maternal and fetal morbidity and mortality (Kuklina, Ayala & Callaghan, 2009; Than et al., 2012). Moreover, women who develop these syndromes are at high risk for metabolic and cardiovascular diseases later in life (Hales & Barker, 2001; Gluckman et al., 2008; Than et al., 2012). Furthermore, in 5–18% of cases affected by these syndromes babies are born preterm with the sequelae of prematurity, low birth-weight and severe neurodevelopmental disorders including cerebral palsy (Moster, Lie & Markestad, 2008; Romero, 2009; Romero, Dey & Fisher, 2014; Mor et al., 2016).

Recent discoveries revealed that a spectrum of obstetrical syndromes ranging from miscarriages through preeclampsia toward stillbirth have overlapping placental pathologies (Brosens et al., 2011). The human placenta differs from that of most mammalian species in that placental trophoblasts deeply invade into the uterus and modify the maternal blood vessels to ensure sufficient and continuous blood flow necessary to supply the developing human fetus with nutrients and oxygen (Brosens et al., 2011). For reasons not yet fully explored, there is frequently a failure of this deep trophoblast invasion in early pregnancy, which leads to premature blood flow to the placenta and placental stress of varying extent, resulting in miscarriages or obstetrical syndromes (Burton & Jauniaux, 2004; Brosens et al., 2011). In other instances, placental functional problems may arise due to reasons other than trophoblast invasion failure, still severely affecting the physiology and immunoregulation of pregnancy (Romero et al., 2006b; Burton et al., 2009).

Since placental developmental and/or functional problems may strongly be reflected by the changed expression of placental proteins (PPs), their examination as biomarkers of pregnancy complications has been in the center of focus of obstetricians and reproductive biologists for decades (Romero et al., 2006a; Erez et al., 2017; Biró & Rigó, 2018). In the pioneering era of placental research, a systematic work started on PPs, including their isolation from the placenta and characterization of their physico-chemical properties (Bohn, 1985; Bohn & Winckler, 1991a, 1991b; Bohn, Winckler & Grundmann, 1991; Than, Bohn & Szabo, 1993). Dr. Hans Bohn was one of the most eminent scientists of the field, who purified more than 50 proteins from the human placenta, which he named sequentially, and generated highly specific antibodies against them (Than, Bohn & Szabo, 1993). Later studies showed that many of the so-called soluble PPs secreted from the placenta into the maternal circulation, such as Placental Protein 5 (PP5, tissue factor pathway inhibitor-2, TFPI-2) or Placental Protein 13 (PP13, galectin-13), have important roles in placental functions and diagnostic significance in pregnancy complications (Than et al., 1999, 2004, 2008, 2011, 2014a, 2014b, 2018; Visegrady et al., 2001; Romero et al., 2008; Balogh et al., 2011; Madar-Shapiro et al., 2018; Karaszi et al., 2019).

Among these purified PPs were also membrane-associated placental proteins (MPs), sequentially named from MP1 to MP10. Some of these MPs have been characterized as already known functional proteins, such as MP1 (heat stable alkaline phosphatase) or MP3 (laminin) that have functional importance in the placenta (Bohn & Winckler, 1991b; Bohn, Winckler & Grundmann, 1991; Than, Bohn & Szabo, 1993). However, the biological properties of many MPs and their placental expression pattern in healthy or disease conditions in pregnancy have remained unknown. Among these was membrane-associated placental protein 4 (MP4), which Dr. Bohn isolated in 1991 (Bohn & Winckler, 1991b). In addition to the placenta, MP4 was detected also in other human tissues (Bohn & Winckler, 1991b; Than, Bohn & Szabo, 1993). The placental localization of MP4 was investigated using immunohistochemistry. In first trimester human placentas, MP4 expression was mainly detected in the basement membrane of extravillous trophoblasts (EVTs), while in term placentas predominant MP4 immunostaining localized to the basement membrane of the villous trophoblasts, the stroma of the villi and the decidual stroma around the EVTs (Than, Bohn & Szabo, 1993). This may suggest that there is a developmental regulation of placental MP4 expression, and that MP4 may be involved in trophoblastic pathologic processes affected in obstetrical syndromes.

Here, our aims were (1) to determine the identity of MP4 with mass spectrometry; and (2) to characterize the placental expression pattern of MP4 in normal and complicated pregnancies, including early pregnancy loss (EPL), preeclampsia and HELLP syndrome, (3) in order to study the potential involvement of MP4 in placental pathologies of these pregnancy complications.

## Materials and Methods

### Study groups, clinical definitions and sample collection

Placental tissue samples were collected from Caucasian women in two studies, processed immediately after sample collection as described before (Hargitai, Marton & Cox, 2004; Than et al., 2008; Varkonyi et al., 2011; Szabo et al., 2013, 2015), fixed in 10% neutral-buffered formalin and were embedded in paraffin (FFPE). In the first study, first trimester placentas (*n* = 44) were collected prospectively at the Maternity Private Clinic of Obstetrics and Gynecology (Budapest, Hungary). Pregnancies were dated according to ultrasound scans and were between 5 and 13 weeks of gestation (GW). Patients with twin gestation or pregnancies with congenital or chromosomal abnormalities were excluded. Women were enrolled in two groups: (1) women who underwent elective termination of pregnancy at their request for non-medical reasons (TOP, *n* = 31, 5–13 GW, first trimester controls); and (2) women with EPL (*n* = 13, 5–13 GW). Cases were matched to controls within one GW. Table 1 contains clinical and demographic information for these study groups.

**Table 1:** Demographic and clinical data of first trimester study groups.

Groups (number of cases^a^)	TOP (*n* = 31)	EPL (*n* = 13)
Maternal age (years)^b^	29 (25–37)	37 (31–39)*
Gestational age at surgery (weeks)^b^	8.71 (7.857–9.857)	9.286 (8.786–10.14)
Gravidity^b^	2 (1–3)	2 (1.5–4)
Parity^b^	0 (0–1)	0 (0–1)
Habitual abortion (two or more consecutive misc.)^c^	3	46**

**Notes:**

All women were Caucasian.

TOP, elective termination of pregnancy, first trimester controls.

EPL, early pregnancy loss.

**p < 0.01; *p < 0.05 compared to gestational age-matched controls.

a Values are presented as number.

b Values are presented as median (interquartile (IQR) range).

c Values are presented as percentage.

Early pregnancy loss was defined as a nonviable, intrauterine pregnancy with a gestational sac containing an embryo or fetus without fetal heart activity within the first 12 6/7 GW according to the American College of Obstetricians and Gynecologists Practice Bulletin (Prager, Dalton & Allen, 2015).

In the second study, samples were collected at the First Department of Obstetrics and Gynecology, Semmelweis University (Budapest, Hungary). Pregnancies were dated according to ultrasound scans between 8 and 12 GW. Patients with twin gestation or fetuses having congenital or chromosomal abnormalities were excluded. Women were enrolled in the following groups: (1) third trimester early controls (*n* = 5, GW ≤ 35); (2) early preeclampsia without HELLP syndrome (*n* = 7, GW ≤ 35); (3) early preeclampsia with HELLP syndrome (*n* = 8, GW ≤ 34); (4) third trimester late controls (*n* = 9, GW ≥ 38); and (5) late preeclampsia (*n* = 8, GW ≥ 36). Cases were matched to controls within two GWs. Table 2 contains clinical and demographic information for these study groups.

**Table 2:** Demographic and clinical data of third trimester study groups.

Groups (number of cases^a^)	Early control (*n* = 5)	Early preeclampsia		Late control (*n* = 9)	Late preeclampsia (*n* = 8)
		Without HELLP sy. (*n* = 7)	With HELLP sy. (*n* = 8)		
Maternal age (years)^b^	31.6 (31.1–34.3)	34.0 (27.6–35)	29.4 (27.1–30.1)	30.8 (30.1–34.2)	31.3 (26–34.2)
Gestational age at delivery (weeks)^b^	31.7 (31–34)	32.6 (31.2–34.4)	29.4 (28.4–32.3)	38.9 (38.7–39.7)	37.4 (36.8–38)^#^
Systolic blood pressure (mm Hg)^b^	120 (120–133)	160 (156.3–160)*	165 (147.5–170)*	130 (125–135)	156.5 (150.8–167.5)^##^
Diastolic blood pressure (mm Hg)^b^	80 (70–80)	100 (100–100)*	100 (97.5–110)*	80 (78–85)	95 (90–100)^##^
Maternal BMI (kg/m^2^)^b^	23.4 (20.1–24.6)	24.4 (23.4–25.2)	24.7 (21.3–26.8)	26.7 (23.1–28)	21.9 (19.6–23.1)
Birthweight (g)^b^	1,990 (910–2,210)	1,100 (1,010–1,280)	965 (885–1,513)	3,470 (3,400–4,030)	2,955 (2,588–3,163)^##^
Placental weight (g)^b^	294 (290–301)	217 (211–227)*	185 (141–279)*	518 (481–650)	470 (431–486)^#^
MVMP score^b^	1 (0–2)	8 (5–8.5)*	6.5 (5–8)**	2 (1–3)	2 (1.75–4.5)
Proteinuria^c^	0	100**	100**	0	100^##^

**Notes:**

All women were Caucasian.

** *p* < 0.01; ^*^p < 0.05 compared to early controls.

## *p* < 0.01; ^#^*p* < 0.05 compared to late controls.

a Values are presented as number.

b Values are presented as median (interquartile (IQR) range).

c Values are presented as percentage.

Preeclampsia was defined according to the criteria set by the American College of Obstetricians and Gynecologists (Gilstrap & Ramin, 2002) and severe preeclampsia was defined according to Sibai, Dekker & Kupferminc (2005). Third trimester early controls had episode of preterm labor leading to preterm birth without clinical or histological signs of chorioamnionitis, they had no other medical complications and delivered a neonate with a birthweight appropriate-for-gestational age (AGA). Third trimester late controls had healthy pregnancy, term delivery and delivered an AGA neonate without medical or obstetrical complications (Papp et al., 1991). Small-for-gestational age was defined as neonatal birthweight below the 10th percentile for gestational age (Papp et al., 1991). Cesarean section was performed in all cases due to severe symptoms as well as in all controls due to previous Cesarean section or malpresentation. HELLP syndrome was specified by hemolysis (serum LDH > 600 IU/L; bilirubin > 1.2 mg/dL; presence of schistocytes in peripheral blood), elevated liver enzymes (serum ALT and/or AST > 70 IU/L) and thrombocytopenia (platelet count < 100,000/mm^3^) (Weinstein, 1982; Barton & Sibai, 2004).

The research was approved by the Health Science Board of Hungary (TUKEB 22-164/2007-1018EKU; 4834-0/2010-1018EKU). Written informed consent was obtained from women prior to sample collection. Clinical samples and data were stored anonymously. The research conformed to the principles set out in the World Medical Association Declaration of Helsinki.

### Mass spectrometry based proteomics

#### Enzymatic digestion of isolated MP4

Lyophilized MP4 protein, isolated by Bohn & Winckler (1991b), was dissolved in 50 mM NH_4_HCO_3_ and the concentration was measured using NanoDrop 2000 spectrophotometer (Thermo Fisher Scientific, Waltham, MA, USA). Then, 2 μL (1.5 μg) was diluted with 8 μL 5% methanol. Proteins were unfolded and reduced using 0.66 μL 0.5% RapiGest SF solution (lyophilized sodium-3-((2-methyl-2-undecyl-1,3-dioxolan-4-yl)-methoxyl)-1-propane-sulfonate, Waters, Milford, MA, USA) and 0.52 μL 200 mM DTT solution (*Fluka* Chemie GmbH; Sigma-Aldrich^®^, Zwijndrecht, Netherlands) for 30 min at 60 °C. Proteins were alkylated using 3.87 μL 200 mM NH_4_HCO_3_ solution and 1.05 μL 200 mM IAA solution (*Fluka* Chemie GmbH; Sigma-Aldrich^®^, Zwijndrecht, Netherlands) for 30 min at room temperature in dark. Digestion was performed by adding 0.5 μL 50 ng/μL Trypsin/Lys-C Mix (Mass Spec Grade; Promega Corporation, Madison, WI, USA) for 60 min at 37 °C, followed by adding 0.5 μL 200 ng/μL Trypsin (Trypsin Gold, Mass Spectrometry Grade; Promega Corporation, Madison, WI, USA) for 120 min at 37 °C. Digestion was stopped by adding 0.5 μL formic acid, followed by 30 min incubation at 37 °C. Samples were centrifuged at 17,000×*g* for 30 min. All other reagents were purchased from Sigma-Aldrich^®^ (St. Louis, MO, USA).

#### Lysis of placental tissue samples

Frozen human placental tissue sample from a normal term healthy pregnancy was placed in liquid nitrogen, pulverized in mortar, and then lysed in two different buffers. Lysis buffer I contained 7 M Urea, 2 M Thiourea, 0.5% RapiGest SF, 30 mM Tris–HCl (pH = 8) and 100 mM DTT. Lysis buffer II was RIPA buffer (Thermo Fisher Scientific, Waltham, MA, USA). In both instances, 66 μL lysis buffer was added to 4–5 mg powdered placental tissue. Samples lysed in Lysis buffer I were sonicated for 10 × 1 min using UP200St-Powerful Ultrasonic Lab Homogenizer (Hielscher Ultrasonics GmbH, Teltow, Germany). Samples lysed in Lysis buffer II were heated for 95 °C for 10 min, sonicated for 3 × 1 min using UP200St-Powerful Ultrasonic Lab Homogenizer, then heated for 95 °C for 10 min again. Concentrations of lysed placental tissue samples were determined with 2D Quant Kit (GE Healthcare, New York, NY, USA). Due to incompatibility of some compounds of Lysis buffer II with tryptic digestion, samples in this buffer were precipitated using 600 μL ice-cold ethanol and then stored at −20 °C overnight. Samples were then centrifuged at 17,000×*g* for 10 min, ethanol was removed, and pellets were washed with 600 μL ice-cold ethanol. After centrifugation at 17,000×*g* for 20 min, ethanol was removed and pellets were solubilized in Solubilization buffer containing 7M Urea and 0.5% RapiGest.

#### Digestion of lysed placental tissue samples

Three to five μL lysed samples containing 25 μg protein were diluted with 50 mM NH_4_HCO_3_ to 20 μL. Samples were reduced using 2 μL 200 mM DTT solution for 1 h at 37 °C. Alkylation was performed using 5 μL 50 mM NH_4_HCO_3_ solution and 4 μL 200 mM IAA solution for 30 min at room temperature in dark. For digestion, 0.5 μL 250 ng/μL Trypsin/Lys-C Mix was used for 60 min at 37 °C, this was followed by treatment with 0.5 μL 1 μg/μL trypsin for 120 min at 37 °C. In other cases 0.5 μL 1 μg/μL chymotrypsin (Chymotrypsin, Sequencing Grade; Promega Corporation, Madison, WI, USA) for 1.5 h at 37 °C was used. At the end of digestion, 1 μL formic acid was added to the samples. After 30 min incubation at 37 °C, samples were centrifuged at 17,000×*g* for 30 min.

#### Cleaning of digested samples

Digested MP4 and placental tissue samples were cleaned using Pierce™ C18 Spin Columns (Thermo Fisher Scientific, Waltham, MA, USA) following the manufacturer’s protocol. However, in case of Sample Buffer and Wash Solution, protocol was modified and 0.1% trifluoroacetic acid was used instead the original buffers. After evaporation with SpeedVac (miVacDuo Concentrator, Genevac Ltd., Ipswich, Suffolk, UK), samples were dissolved in 30 μL 0.1% formic acid.

#### HPLC-MS/MS analysis

Analysis of the digested samples was performed using HPLC-MS/MS. Chromatographic separation was performed using nanoflow UHPLC system, which was coupled to a high resolution QTOF mass spectrometer (Dionex UltiMate 3000 RSLCnano System, Thermo Scientific, Sunnyvale, CA, USA, Maxis II ETD, Bruker Daltonik GmbH, Bremen, Germany) equipped with a CaptiveSpray nanoBooster ionization source. NanoBooster was filled with acetonitrile, nitrogen pressure was 0.2 bar. Acclaim Pepmap C18 trap column (100 μm i.d. × 20 mm) was used for on-line desalting of samples, and a reverse phase Acclaim Pepmap RSLC analytical column (C18, 75 μm i.d. × 250 mm) was used for the separation of peptides. Elution was performed using a 113 min long gradient—from 3% to 30% solvent B—and a flow rate of 300 nL/min. Solvent A was LC-MS grade water containing 0.1% formic acid and solvent B was LC-MS grade acetonitrile also containing 0.1% formic acid. Column temperature was set to 48 °C.

CaptiveSpray ionization mass spectrometric analysis was performed in positive mode. Signal intensities were measured using single stage mass spectrometry, and protein identification was performed using tandem mass spectrometry in AutoMSMS setup (data dependent analysis). Parameters were the following: capillary voltage: 1.2 kV, drying gas flow: 3 L/min, gas temperature: 150 °C, mass range: 150–2,200 *m/z*, for fragmentation of peptides CID mode was used–applied collision gas was N_2_, collision energy was 10 eV. In case of glycoproteomic studies mass range was 150–3,000 *m/z* and collision energy was 7 eV.

Proteomic data evaluation was performed with ProteinScape 4.0.3 software (Bruker Daltonik GmbH, Bremen, Germany) using Mascot 2.5.1 search engine (Matrix Science, London, UK). Data were searched against SwissProt 2015_08 database (549,008 sequences), and searching was performed on 20,204 human sequences. Two missed cleavages were allowed, carbamidomethyl C was set as fixed modification, deamidation (NQ) and oxidation (M) were set as variable modifications. Peptide tolerance was 7 ppm, MS/MS tolerance was 0.05 Da—based on monoisotopic masses. 1+, 2+ and 3+ charged states of peptides were searched, results were accepted below 1% FDR and Percolator was used. *N*-glycans were searched against the Consortium for Functional Glycomics *Homo sapiens* glycan database including HexNAc 2-5, Hex 3-12, NeuAc 0-4 and Fuc 0-1 compositions. Charge state was set from 1+ to 6+, MS and MS/MS tolerance were the same as during peptide search.

Glycoproteomic data evaluation was also performed using Byonic™ and Preview™ (Protein Metrics Inc., San Carlos, CA, USA) software. Preview was used to determine the optimal searching parameters with the following parameters: carbamidomethyl C as fixed modification and trypsin and chymotrypsin with semi specific search; UniProt SwissProt *Homo sapiens* protein database (160,363 sequences). Initial Byonic search parameters were augmented with *N*-glycan search of 309 mammalian glycans.

### Histopathologic evaluation of the placentas

Five μm sections were cut from FFPE tissue blocks of first and third trimester placentas and stained with hematoxylin and eosin for histopathological evaluation at the 1st Department of Pathology and Experimental Cancer Research, Semmelweis University. The sections were examined using light microscopy by a perinatal pathologist blinded to the clinical information. Histopathologic changes were defined according to published criteria (Hargitai, Marton & Cox, 2004; Redline, 2008; Than et al., 2008; Varkonyi et al., 2011; Khong et al., 2016). In case of third trimester placentas, maternal vascular malperfusion (MVMP) score was created and summarized according to a previous study (Than et al., 2018).

### Tissue microarray construction, perlecan immunostaining

As previously described (Hargitai, Marton & Cox, 2004; Varkonyi et al., 2011; Szabo et al., 2013, 2015), representative areas were selected for the construction of Tissue microarrays (TMAs), which contained 2 mm cores in diameter. To investigate the protein expressions in early and late pregnancy, five TMAs were created. Two TMAs were created using an automated tissue arrayer (TMA Master II, 3DHISTECH, Budapest, Hungary) to contain one block of each first trimester placenta (*n* = 44) in triplicate. Three TMAs were created to contain four-five tissue blocks of each third trimester placenta (*n* = 37).

Five μm sections were cut from TMAs and were placed on silanized slides. After deparaffinization and rehydration, endogen peroxidase blocking was performed using 10% H_2_O_2_ for 20 min. Antigen retrieval was performed using Tris EDTA buffer (10 mM Tris; 1 mM EDTA; 0.05% Tween 20; pH = 9) for 3 min at 100 °C in pressure cooker, then using Bond™ Enzyme Pretreatment Kit (AR9551, Leica Biosystems Newcastle Ltd, enzyme ½—half drop of enzyme in 7 mL buffer) at 37 °C for 5 min. Immunostaining was carried out using Novolink Polymer Detection System (Novocastra Laboratories, Newcastle, UK, Peroxidase/DAB+, Rabbit), according to the manufacturer’s protocol. Slides were blocked for 10 min with Protein Block.

We performed immunostainings with different conditions to examine first and third trimester perlecan expression due to its gestational age dependence. (1) To compare perlecan expression in first and third trimester placentas, slides were incubated with anti-perlecan mouse monoclonal antibody (Thermo Fisher Scientific, Waltham, MA, USA) at 1:60 dilution in 1% BSA-TBS overnight at 4 °C. (2) To analyze perlecan expression among first trimester patient groups, slides were incubated with anti-perlecan antibody at 1:60 dilution. (3) To analyze perlecan expression among third trimester patient groups, slides were incubated with anti-perlecan antibody at 1:30 dilution. (4) By technical negative control the primary antibody was omitted. In all circumstances, following steps were the same. Briefly, after three washes with TBST and Post Primary treatment (30 min, at room temperature), Novolink Polymer was used as secondary antibody for 30 min at room temperature. This was followed by three washes with TBST, and then sections were developed using 3,3′-diaminobenzidine (Novolink) at 1:20 dilution. Finally, sections were counterstained with hematoxylin, and these were mounted (DPX Mountant; Sigma-Aldrich, St. Louis, MO, USA) after dehydration.

### Evaluation of immunostainings

Perlecan immunostained placental TMAs were digitally scanned by a high-resolution bright field slide scanner (Pannoramic Scan; 3DHistech Ltd., Budapest, Hungary) and cytoplasmic staining in the syncytiotrophoblast was evaluated blinded to the clinical information on virtual slides using Pannoramic Viewer 1.15.4 (3DHistech Ltd., Budapest, Hungary). In first trimester TMAs, all villi were scored semi-quantitatively. In third trimester TMAs, at least 25 terminal or intermediate villi (diameter of 20–150 μm) were scored semi-quantitatively. Due to the difference in perlecan expression between first and third trimesters, we used separate scoring systems for each comparisons, which were both based on a previously published analysis (Than et al., 2008). The intensity of immunostaining was graded as 0 to +3. The average intensity was determined for each core as the representative data for that core. By averaging immunoscores of the cores, the overall intensity score was assigned to each patient group. The correlations between perlecan immunoscores and MVMP score of the placenta, placental weight or birthweight were also investigated.

### Data analysis

Demographic data was analyzed using Microsoft Excel version 2016 (Microsoft Corp., Redmond, WA, USA) and GraphPad Prism 7 (GraphPad Software, La Jolla, CA, USA). Comparisons among the groups were performed using Chi-square and Fisher’s exact tests for proportions, Kruskal–Wallis and Mann–Whitney tests for non-normally distributed continuous variables, and the Student’s *t*-test for normally distributed continuous variables. Statistical significance was considered at *p* < 0.05.

The Human Protein Atlas database (www.proteinatlas.org) and the GeneCards (www.genecards.org) records were used to analyze the tissue localization and expression of perlecan in the human placenta. The expression of perlecan in the EVT and villous trophoblast (STB) lineages was studied by retrieving and reanalyzing an earlier published microarray dataset (accession number: GSE9773) (Bilban et al., 2009). To study the potential changes in perlecan expression in the placenta with gestational age, we reanalyzed gene expression data from gene expression omnibus (GEO) (accession number: GSE9984) (Mikheev et al., 2008). Microarray data were plotted with “R” (www.r-project.org) as boxplot.

## Results

### Demographic and clinical data

Demographic and clinical characteristics of patients in the two studies are displayed in Tables 1 and 2. In the first study, maternal age was higher in EPL than in first trimester controls (TOP). The prevalence of habitual abortion was higher in cases of miscarriages (EPL, 46%) than in controls (TOP, Table 1). In the second study, systolic and diastolic blood pressures were higher in the groups with early preeclampsia with or without HELLP syndrome. Proteinuria was detected in all cases except control groups. Although third trimester early and late controls were matched to cases within two weeks of gestational age, the median gestational age of third trimester late controls was slightly higher than that of cases with late preeclampsia. Birthweight was lower in late preeclampsia than in controls, and they tended to be lower in early preeclampsia with or without HELLP syndrome than in their respective controls. Placental weight was lower in all disease groups compared to controls. MVMP score was higher in early preeclampsia with and without HELLP syndrome compared to early controls (Table 2).

### Mass spectrometry based proteomics

#### Proteomic identification of MP4

MP4 purified from placenta was identified by mass spectrometry based proteomics as basement membrane-specific heparan sulfate proteoglycan core protein (UniProt identifier: PGBM_HUMAN; Table 3). This protein is also known as perlecan, and it is expressed by *HSPG2* gene located on chromosome 1 (Murdoch et al., 1992). Protein identification was reliable with 137 peptides based on MS/MS fragmentation and 30.9% sequence coverage.

**Table 3:** Identified proteins in purified membrane-associated placental protein 4 (MP4).

Row	Accession	Protein	MW (kDa)	Mascot scores	#Peptides	Sequence coverage (%)
1	PGBM_HUMAN	Basement membrane-specific heparan sulfate proteoglycan core protein OS=Homo sapiens GN=HSPG2 PE=1 SV=4	468.5	4,779.8	137	30.9
2	TINAL_HUMAN	Tubulointerstitial nephritis antigen-like OS=Homo sapiens GN=TINAGL1 PE=1 SV=1	52.4	395.1	13	31.7
3	FBLN2_HUMAN	Fibulin-2 OS=Homo sapiens GN=FBLN2 PE=1 SV=2	126.5	277.2	8	7.3
4	NID2_HUMAN	Nidogen-2 OS=Homo sapiens GN=NID2 PE=1 SV=3	151.2	224.1	7	8.1
5	FBLN1_HUMAN	Fibulin-1 OS=Homo sapiens GN=FBLN1 PE=1 SV=4	77.2	211.0	7	9.5
6	COCA1_HUMAN	Collagen alpha-1(XII) chain OS=Homo sapiens GN=COL12A1 PE=1 SV=2	332.9	196.6	7	3.6
7	IGHG1_HUMAN	Ig gamma-1 chain C region OS=Homo sapiens GN=IGHG1 PE=1 SV=1	36.1	176.7	7	25.5
8	TRFE_HUMAN	Serotransferrin OS=Homo sapiens GN=TF PE=1 SV=3	77	168.6	7	9.6
9	IGHG3_HUMAN	Ig gamma-3 chain C region OS=Homo sapiens GN=IGHG3 PE=1 SV=2	41.3	154.0	6	16.2
10	ALBU_HUMAN	Serum albumin OS=Homo sapiens GN=ALB PE=1 SV=2	69.3	138.5	5	10.7
11	NID1_HUMAN	Nidogen-1 OS=Homo sapiens GN=NID1 PE=1 SV=3	136.3	127.4	5	5.2
12	FINC_HUMAN	Fibronectin OS=Homo sapiens GN=FN1 PE=1 SV=4	262.5	125.4	6	3
13	PRG2_HUMAN	Bone marrow proteoglycan OS=Homo sapiens GN=PRG2 PE=1 SV=2	25.2	87.9	4	21.6
14	H2B1K_HUMAN	Histone H2B type 1-K OS=Homo sapiens GN=HIST1H2BK PE=1 SV=3	13.9	69.4	2	19
15	ACTB_HUMAN	Actin, cytoplasmic 1 OS=Homo sapiens GN=ACTB PE=1 SV=1	41.7	65.7	2	5.3
16	VTNC_HUMAN	Vitronectin OS=Homo sapiens GN=VTN PE=1 SV=1	54.3	59.9	2	4.6
17	VWA1_HUMAN	von Willebrand factor A domain-containing protein 1 OS=Homo sapiens GN=VWA1 PE=2 SV=1	46.8	55.1	2	2.9
18	NFH_HUMAN	Neurofilament heavy polypeptide OS=Homo sapiens GN=NEFH PE=1 SV=4	112.4	43.2	2	1.7
19	HORN_HUMAN	Hornerin OS=Homo sapiens GN=HRNR PE=1 SV=2	282.2	43.1	2	1.3
20	ENOA_HUMAN	Alpha-enolase OS=Homo sapiens GN=ENO1 PE=1 SV=2	47.1	42.1	2	6.2
21	FIBG_HUMAN	Fibrinogen gamma chain OS=Homo sapiens GN=FGG PE=1 SV=3	51.5	37.3	2	4.9
22	MYH9_HUMAN	Myosin-9 OS=Homo sapiens GN=MYH9 PE=1 SV=4	226.4	34.0	2	1.3

After the identification of purified MP4/perlecan, we tested two lysis conditions for the analysis of normal term placental tissue, as well. We identified MP4/perlecan in Lysis buffer I, utilizing urea- and RapiGest-based lysis buffer with high confidence (70 peptides, 25.6% sequence coverage). However, in Lysis buffer II, which utilized RIPA buffer, MP4/perlecan was under the limit of detection.

#### Characterization of N-glycans on purified and placental perlecan

Ten potential *N*-glycosylation sites are listed for perlecan in the UniProt database (www.uniprot.org). Out of this 10, our glycoproteomic analysis identified four sites to be glycosylated on the protein backbones of both the purified and placental tissue perlecan. Using trypsin and LysC enzymes during digestion, we found glycans on Asn89, Asn1755 and Asn3780. After digestion with chymotrypsin Asn4068 was found to be glycosylated, as well.

**Figure 1:**
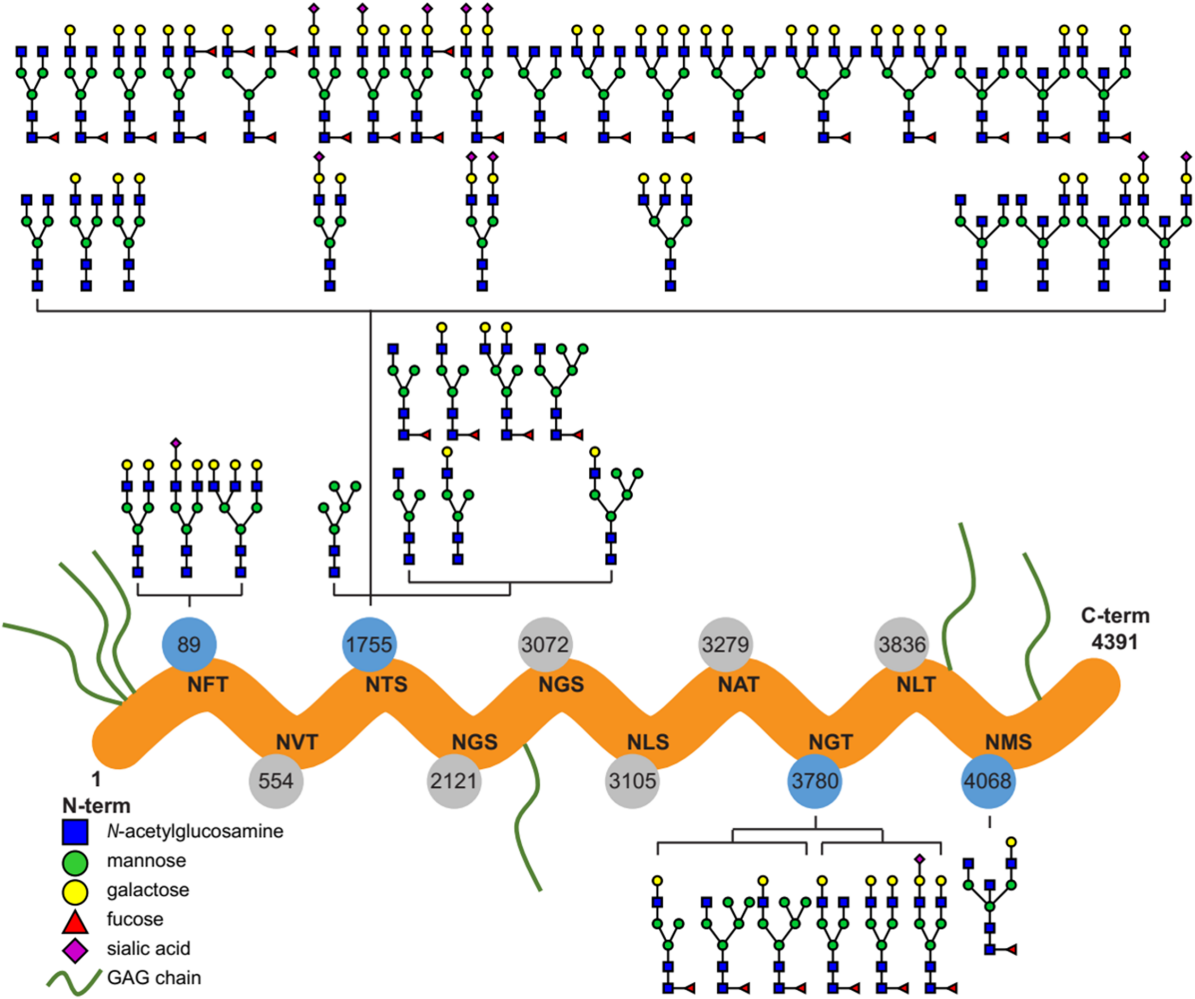
Overview of the site-specific *N*-glycan distribution of perlecan. The *N*-glycosylation microheterogeneity at the individual sites of the perlecan amino acid sequence is shown. Identified glycosylated residues with Asn-X-Ser/Thr motif are colored in blue, whereas non-identified residues are labeled in gray. Glycosaminoglycan chains are abbreviated as GAG chains.

Detailed analysis of the identified *N*-glycans showed ∼90% complex type glycans on the protein in case of both the purified and placental samples. Glycan structures identified by the software, based on the mass spectra, were manually filtered and curated (Fig. 1; Table S1).

### Perlecan expression in normal placentas throughout gestation

**Figure 2:**
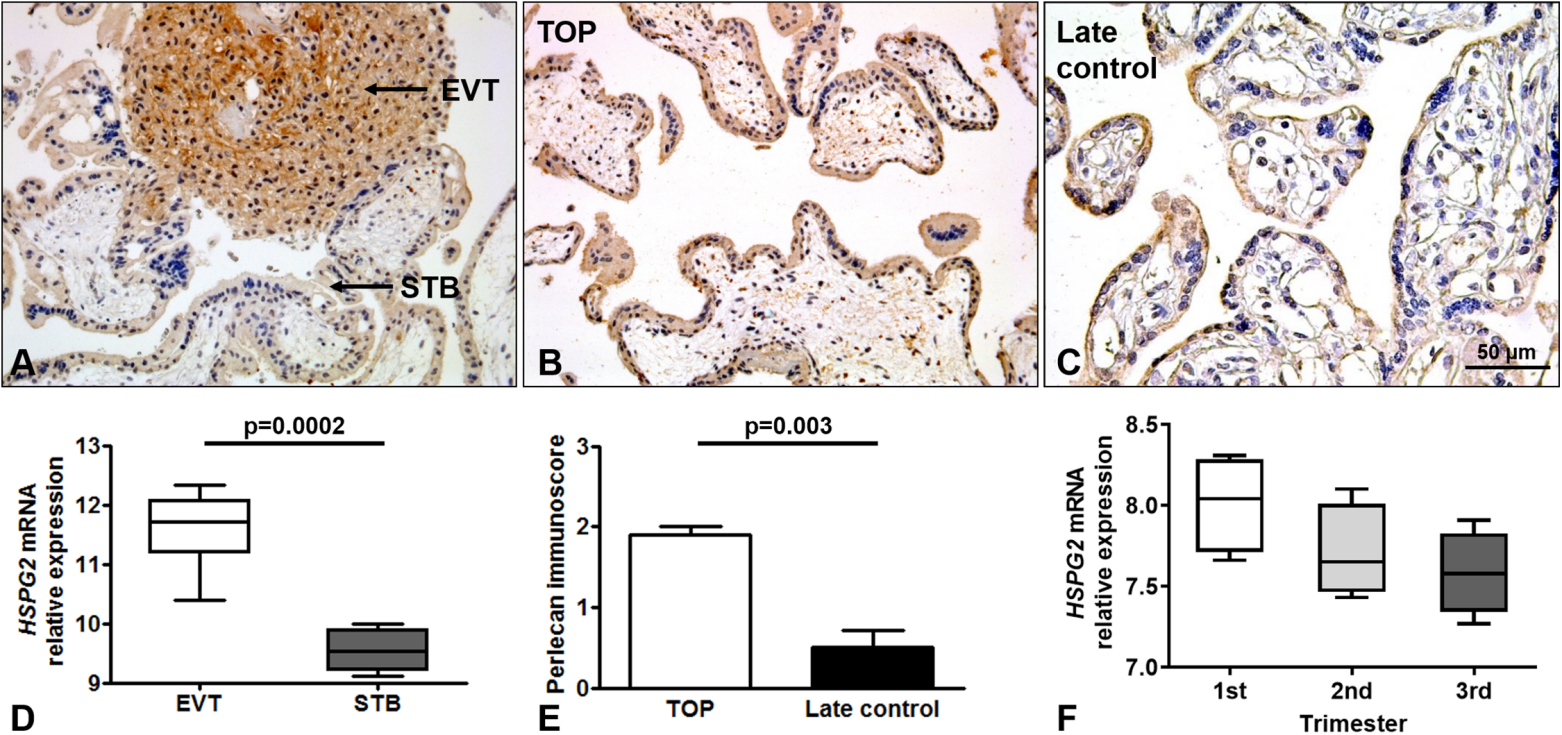
Perlecan expression in first and third trimester placental tissues. (A) Perlecan immunostaining was detected in the syncytiotrophoblast and cytotrophoblasts of first trimester placenta (GW9). There was a stronger staining in extravillous trophoblast (EVT) than in the syncytiotrophoblast (STB). (B and C) Representative images show stronger syncytiotrophoblastic perlecan expression in first trimester control (TOP) ((B) GW9) compared to third trimester late control ((C) GW40) placentas. Representative images, hematoxylin counterstain, 200× (A and B) and 400× (C) magnification, scale bar: 50 μm. (D) Box-plots represent gene expression levels of *HSPG2* gene encoding perlecan in EVT and STB from first trimester placental tissue. Data was derived from GEO database (GSE9773). (E) Syncytiotrophoblastic perlecan immunoscore was higher in some selected placentas collected from first trimester controls (TOP: mean: 1.91 ± SE: 0.09, *p* = 0.003) than in third trimester late controls (mean: 0.5 ± SE: 0.21). (F) Box-plots represent gene expression levels in placentas from all trimesters. Data was derived from GEO database (GDS4037).

Perlecan expression was detected by immunohistochemistry of first and third trimester placental tissue specimens. In the first trimester, a stronger perlecan immunostaining was detected in the extravillous trophoblasts than in the villous trophoblasts of the chorionic villi (Fig. 2A). Stronger perlecan immunostaining was observed in the cytoplasm of the syncytiotrophoblast in first trimester controls (TOP, Fig. 2B) than in third trimester late controls (Fig. 2C). Indeed, we found higher perlecan immunoscore in some selected placentas in first trimester controls (TOP, mean: 1.91 ± standard error: 0.09) than in third trimester late controls (0.5 ± 0.21, *p* = 0.003, Fig. 2E).

Immunohistochemical data are supported by GEO records, as perlecan mRNA expression was higher in the extravillous trophoblast than in the syncytiotrophoblast in first trimester (Fig. 2D), and decreased with gestational age in placental tissue (Fig. 2F).

### Placental perlecan expression in miscarriages

**Figure 3:**
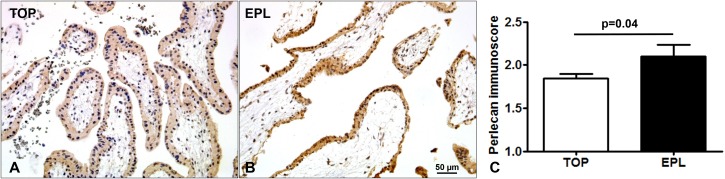
Placental perlecan immunostaining in first trimester miscarriages. Syncytiotrophoblastic perlecan immunostaining was stronger in early pregnancy loss ((B) EPL, GW9) compared to first trimester controls ((A) TOP, GW9). (C) Perlecan immunoscores were higher in EPL (mean: 2.1 ± SE: 0.13, *p* = 0.040, *n* = 13) than in first trimester controls (TOP: 1.84 ± 0.06, *n* = 31). Representative images, hematoxylin counterstain, 200× magnifications, scale bar: 50 μm.

Immunostaining of TMAs allowed the simultaneous examination of perlecan expression in 44 first trimester placentas (Fig. 3). There was a stronger syncytiotrophoblastic perlecan immunostaining in EPL (Fig. 3B) compared to first trimester controls (TOP, Fig. 3A). Accordingly, perlecan immunoscores were higher in EPL than in first trimester controls (EPL: 2.1 ± 0.13, TOP: 1.84 ± 0.06, *p* = 0.040, Fig. 3C).

### Placental perlecan expression in preeclampsia and HELLP syndrome

**Figure 4:**
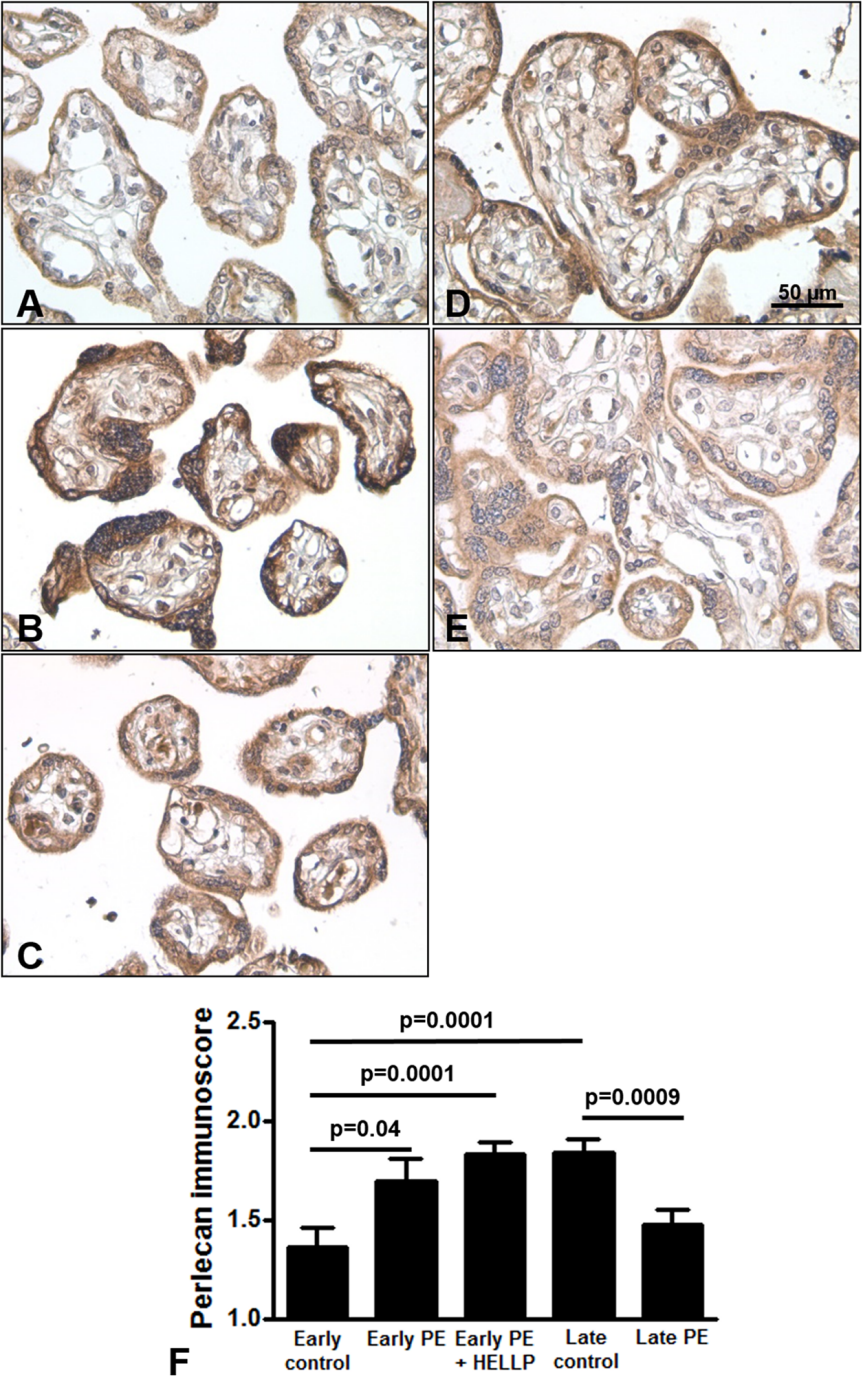
Placental perlecan expression in preeclampsia with or without HELLP syndrome. (A–E) Perlecan immunostaining of the syncytiotrophoblast was stronger in early preeclampsia without HELLP syndrome ((B) GW34) and with HELLP syndrome ((C) GW34) than in third trimester early controls ((A) GW34). Weaker syncytiotrophoblastic perlecan immunostaining was detected in late preeclampsia ((E) GW 40) than in third trimester late controls ((D) GW39). (F) Syncytiotrophoblastic perlecan immunoscores increased with gestational age within controls (third trimester early controls mean: 1.37 ± SE: 0.09, *n* = 5 vs. third trimester late controls: 1.84 ± 0.07, *p* = 0.0001, *n* = 9). Syncytiotrophoblastic perlecan immunoscores were higher in early preeclampsia with and without HELLP syndrome (early PE: 1.69 ± 0.12, *p* = 0.040, *n* = 7; early PE + HELLP: 1.83 ± 0.06, *p* = 0.0001, *n* = 8) than in third trimester early controls (1.37 ± 0.09). Perlecan immunoscores were lower in late preeclampsia (1.47 ± 0.08, *p* = 0.0009, *n* = 8) than in third trimester late controls (1.84 ± 0.07). PE: preeclampsia; HELLP: HELLP syndrome; PE + HELLP: preeclampsia with HELLP syndrome, *p < 0.05, SE: standard error of mean.

Next, we analyzed whether perlecan expression is altered in third trimester pregnancy complications. Here, we used lower dilution of the antibody to enable better visualization of perlecan expression in third trimester placentas, which resulted in higher mean immunoscores for the third trimester late control group. Perlecan immunostaining of 37 third trimester placentas showed predominantly cytoplasmic staining of the syncytiotrophoblast (Fig. 4). Perlecan immunoscores of the syncytiotrophoblast increased with gestational age within third trimester placentas (third trimester early controls: 1.37 ± 0.09 vs. third trimester late controls: 1.84 ± 0.07, *p* = 0.0001) (Figs. 4A, 4D and 4F). Also, there was higher syncytiotrophoblast immunoscore in early preeclampsia with or without HELLP syndrome compared to gestational age-matched controls (early preeclampsia: 1.69 ± 0.12, *p* = 0.040; early preeclampsia with HELLP syndrome: 1.83 ± 0.06, *p* = 0.0001; third trimester early controls: 1.37 ± 0.09) (Figs. 4A–4C and 4F). However, there was lower syncytiotrophoblast immunoscore in late preeclampsia (1.47 ± 0.08, *p* = 0.0009) compared to third trimester late controls (1.84 ± 0.07) (Figs. 4D–4F).

**Figure 5:**
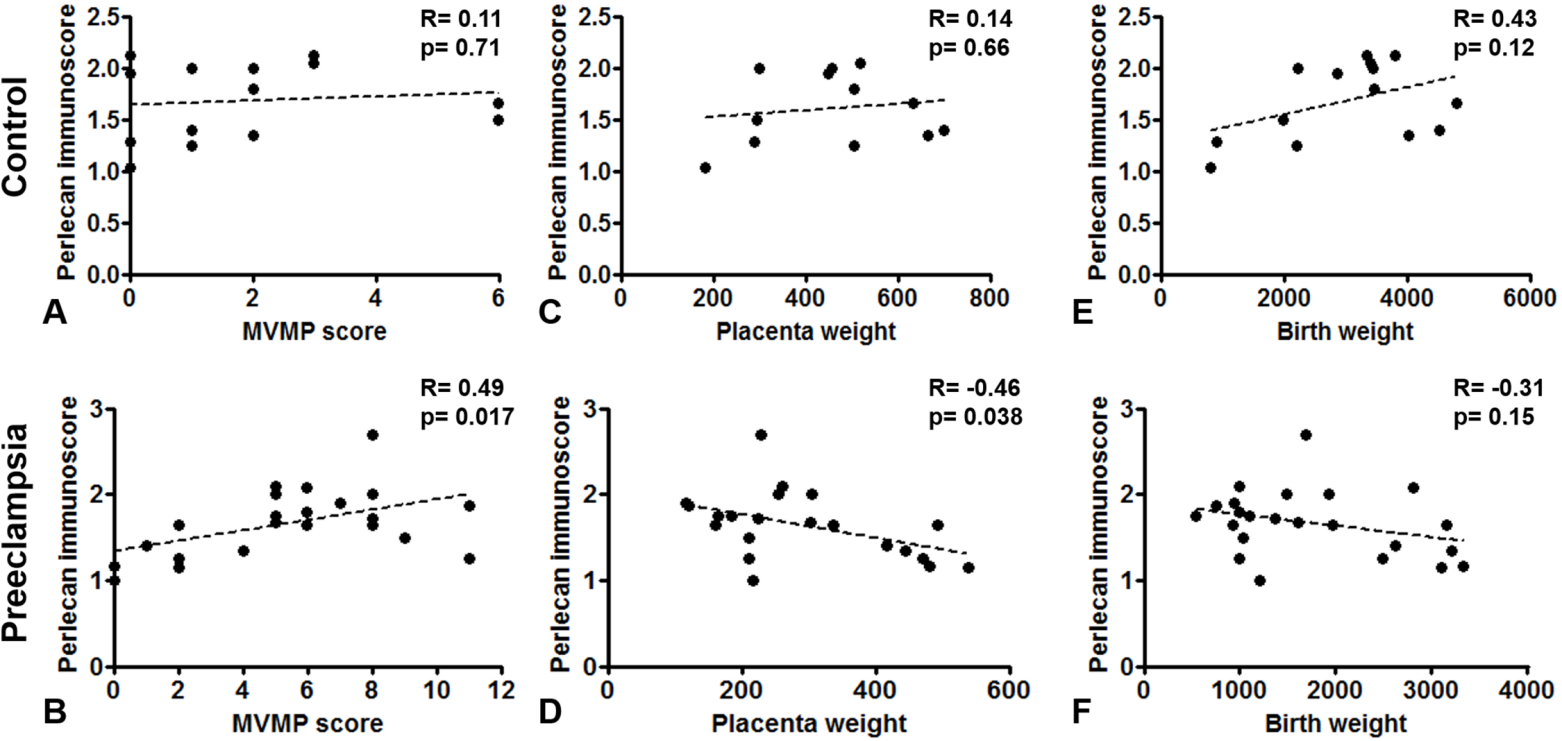
Correlation of perlecan immunoscores with clinical parameters. (A and B) Perlecan immunoscore positively correlated with maternal vascular malperfusion (MVMP) score of the placenta in preeclampsia ((B) *R* = 0.49, *p* = 0.017) but not in controls ((A) *R* = 0.11, *p* = 0.707). (C and D) Furthermore, perlecan immunoscore negatively correlated with placental weight among patients with preeclampsia ((D) *R* = −0.46, *p* = 0.038), but this was not significant in the control group ((C) *R* = 0.14, *p* = 0.653). (E and F) Perlecan immunoscore tended to negatively correlate with birthweight among patients with preeclampsia ((F) *R* = −0.31, *p* = 0.152), but this was not significant among controls ((E) *R* = 0.43, *p* = 0.12).

Perlecan immunoscore positively correlated with placental MVMP score of the placenta in all patients with preeclampsia (*R* = 0.49, *p* = 0.017), but not in controls (*R* = 0.11, *p* = 0.707, Figs. 5A and 5B). Perlecan immunoscore negatively correlated with placental weight among patients with preeclampsia (*R* = −0.46, *p* = 0.038), but this was not significant among controls (*R* = 0.14, *p* = 0.653, Figs. 5C and 5D). Moreover, perlecan immunoscore tended to negatively correlate with birthweight among patients with preeclampsia (*R* = −0.31, *p* = 0.152, Figs. 5E and 5F).

## Discussion

### Principal findings of this study

(1) MP4 was identified as basement membrane-specific heparan sulfate proteoglycan core protein, also known as perlecan; (2) Perlecan was localized to the trophoblast by immunohistochemistry and perlecan immunostaining was stronger in extravillous trophoblasts than in the syncytiotrophoblast in first trimester placentas; (3) There was a decreasing tendency in placental perlecan expression at both mRNA and protein levels throughout gestation; (4) Perlecan immunostaining of the syncytiotrophoblast was stronger in EPL than in first trimester controls (TOP); (5) There was a stronger perlecan immunostaining of the syncytiotrophoblast in early preeclampsia with or without HELLP syndrome than in third trimester early controls. However, decreased perlecan staining was observed in late preeclampsia compared to third trimester late controls; and (6) in preeclampsia, perlecan immunoscores positively correlated with the MVMP score of the placenta, while negatively correlated with placental weight.

### MP4 was identified as perlecan

MP4 was discovered in 1991 by Bohn & Winckler (1991b), who found MP4 only in the urea extract fraction but not in the saline or Triton-X extracts of the placenta (Bohn & Winckler, 1991b). Our results well correspond with Bohn’s data as we could identify MP4 in human healthy placental tissue only using Lysis buffer I containing urea, which is a much stronger buffer than Lysis buffer II (RIPA buffer).

The estimated average amount of MP4 in one placenta was found to be 24 mg by Bohn & Winckler (1991b). The electrophoretic mobility of MP4 was shown to be between ß1- and ß2-globulins, and MP4 was found to consist of several subunits. The average sialic acid content of MP4 determined by neuraminidase treatment was 0.45 weight percent. Subsequently, a specific antiserum was developed for MP4 as for other MPs by immunizing rabbits with the solubilized protein fractions of the human placenta (Bohn & Winckler, 1991b). This antibody was used to characterize body-wide expression pattern of MP4 using gel diffusion test by Bohn, Winckler & Grundmann (1991; Bohn & Winckler, 1991b; Than, Bohn & Szabo, 1993) and they found it in several tissues.

Our study has confirmed many aspects what was described by Bohn et al. For the first time, we identified MP4 purified from placenta as basement membrane-specific heparan sulfate proteoglycan core protein, also called as perlecan. The theoretical molecular weight of perlecan core without any post-translational or amino acid modifications, calculated from the amino acid sequence, is ∼470 kDa. Perlecan has five molecular domains, a heparan sulfate attachment domain, a low-density lipoprotein (LDL) receptor-like domain, two different laminin-like domains and a neural cell adhesion molecule (N-CAM)-like domain (Murdoch et al., 1992; Noonan & Hassell, 1993). Since, perlecan is a large proteoglycan, technical limitations due to its size inhibited the determination of the crystal structure of the entire molecule, and only crystal structures of the laminin-like globular domain (Le et al., 2011) and the immunoglobulin type N-CAM domain (Kvansakul et al., 2001) could be determined. Perlecan is an integral membrane protein, without having any transmembrane domain, and this protein is a major component of the basement membranes in many tissues (Farach-Carson & Carson, 2007; Farach-Carson et al., 2014; Jakab, 2018). It is a secreted extracellular matrix member of heparan sulfate proteoglycans, which consist of a core protein and one or more covalently attached heparan sulfate sidechains (Sarrazin, Lamanna & Esko, 2011). With its heparan sulfate chains, perlecan can bind numerous biologically important molecules such as growth factors, integrins and other basement membrane proteins. Therefore, heparan sulfate chains are critical for the biological functions of perlecan, including providing integrity for the basement membranes and affecting cellular growth, adhesion and invasion (Knox & Whitelock, 2006; Chen et al., 2008).

In spite of that glycosylation is one of the most important post-translational modifications of proteins affecting a wide variety of biological functions (Varki, 1993; Dwek, 1995), functional studies on perlecan have not yet revealed any effect of *N*-glycosylation on this proteoglycan (Knox & Whitelock, 2006; Farach-Carson et al., 2014). As the first step in the understanding of the role of *N*-glycans in perlecan’s placental functions in pregnancy, we characterized *N*-glycosylation sites, types and the structure and distribution of *N*-glycans on perlecan core protein. We identified four *N*-glycosylation sites on the perlecan protein core (Asn89, Asn1755, Asn3780, Asn4068) and found mostly complex type *N*-glycans (∼90%, Fig. 1). Based on UniProt data, perlecan has 10 potential *N*-glycosylation sites (Asn89, Asn554, Asn1755, Asn2121, Asn3072, Asn3105, Asn3279, Asn3780, Asn3836, Asn4068). Among them seven (Asn554, Asn1755, Asn2121, Asn3072, Asn3780, Asn3836, Asn4068) was found to be glycosylated in different studies using samples of different origins and methods (Zhang et al., 2003; Liu et al., 2005; Chen et al., 2009). However, none of these publications analyzed the structure of glycans, only identified the glycosylation sites. It could be interesting to see whether perlecan also undergoes similar glycosylation changes in the placenta during normal pregnancy (Lee & Damjanov, 1984; Nemansky et al., 1998; Robajac et al., 2014) and in pregnancy complications as other proteins (Robajac et al., 2016). However, this would need to be investigated in a separate study.

### Perlecan placental expression is decreased throughout gestation

After the identification of perlecan core protein, we aimed to examine the behavior of this protein in the placenta in normal and pathological conditions throughout gestation since perlecan was previously shown to be involved in cellular functions that are key in placental physiology (Chen et al., 2008; Chang & Vivian Yang, 2013; Oliveira et al., 2015). Interestingly, perlecan was first described in the basement membranes of various normal and tumorous human tissues where it regulated cell attachment and invasion (Hassell et al., 1980; Murdoch et al., 1994), processes also key in placental development. Later, perlecan expression was found on the apical surface of the trophectoderm of the mouse blastocyst, which suggests that perlecan may be involved in embryo development and implantation (Carson, Tang & Julian, 1993). In our study, we observed stronger perlecan immunostaining in the cytoplasm of the syncytiotrophoblast in first trimester controls (TOP) than in third trimester late controls. This is consistent with placental perlecan mRNA expression data downloaded from GEO that showed the same decrease with gestational age. Moreover, Yang et al. (2005) also showed decreasing placental perlecan expression with advancing gestational age at mRNA and protein levels. This further substantiates the developmental regulation of perlecan expression in the placenta and may suggest its regulatory role in first trimester placental development.

In this context, it is important that we found stronger perlecan protein expression in the extravillous trophoblast than in the syncytiotrophoblast in the first trimester, consistent with previous studies showing perlecan mainly in the extravillous trophoblast (Mühlhauser et al., 1996; Yang et al., 2005). This indicates that perlecan may be a factor regulating the functions including the invasive properties of extravillous trophoblasts, in accord with the key role of basement membrane proteoglycans in regulating cell invasion (Wight, Kinsella & Qwarnström, 1992; Sanderson, 2001). Of importance, extravillous trophoblast invasion into the decidua and maternal spiral arteries is critical to the development of proper uteroplacental circulation (Redman & Sargent, 2005), which is inhibited in various obstetrical syndromes including preeclampsia, preterm labor or IUGR (Brosens et al., 2011). The question whether the altered expression of proteoglycans including perlecan in extravillous trophoblasts may be upstream to abnormal trophoblast invasion or just a consequence in these syndromes. In this regard, knock out studies in mice showed that dystroglycan (Williamson et al., 1997) is pivotal in embryogenesis and regulates trophoblast invasion (Street et al., 2012). Knockout studies of nidogen, agrin and perlecan in mice also showed some but not lethal effects (Pozzi, Yurchenco & Iozzo, 2017). However, since trophoblast invasion is very limited in mice (Carter, 2007; Grigsby, 2016), these studies were not able to clarify the role of perlecan or other basement proteoglycans in trophoblast invasion in human. Maybe a non-human primate model would be better suited for this study.

### Placental perlecan expression is increased in early pregnancy loss and in early preeclampsia with HELLP

Abnormal trophoblast invasion and plugging of the maternal spiral arteries is a phenomenon mainly detectable in preeclampsia and miscarriages with a larger extent in the latter (Burton & Jauniaux, 2004; Jauniaux & Burton, 2005; Jauniaux, Poston & Burton, 2006). This leads to a premature blood flow, ischaemia-reperfusion injury and oxidative stress of the placenta, causing embryotoxic effects in miscarriages and placental malfunction and maldevelopment in preeclampsia (Jauniaux et al., 2000; Burton & Jauniaux, 2004; Jauniaux & Burton, 2005; Jauniaux, Poston & Burton, 2006). Because of the known, important roles of basement membrane proteoglycans in invasion (Wight, Kinsella & Qwarnström, 1992; Sanderson, 2001) and the regulation of expression of perlecan under hypoxic condition (Asplund et al., 2010) we hypothesized that perlecan may have expressional changes both in miscarriages and preeclampsia either as a cause or consequence of the common pathology in these syndromes. To investigate this hypothesis, we immunostained five TMAs constructed from 81 placentas obtained from miscarriages, various preeclampsia subtypes as well as first or third trimester control cases. Since our placental specimen collection mainly contained chorionic villous tissue, we could focus our investigation of perlecan expression only on villous but not extravillous trophoblasts. This limited our study to the testing of the association of perlecan expression with hypoxic-ischaemic injury of the placenta.

We found elevated perlecan expression in the syncytiotrophoblast in miscarriages compared to first trimester controls collected from elective termination of pregnancy (TOP). Since the habitual abortion rate was higher in our EPL group than among the average EPL patient population, our results may reflect the expressional changes of perlecan in habitual abortion rather than in a general EPL group. Similarly, higher perlecan expression in the syncytiotrophoblast was detected in early preeclampsia coupled with or without HELLP syndrome than in gestational age-matched controls. However, in late preeclampsia different alteration of perlecan expression was found. This is remarkable since the pathology of early preeclampsia originates from above described abnormal trophoblast invasion-ischaemic injury-placental stress, while late preeclampsia is rooted in a different pathology (Barton & Sibai, 2004; Than et al., 2008, 2014a).

To further investigate the connection between placental perlecan expression and ischaemic placental injury, we correlated perlecan immunoscores with the MVMP score of the placenta. The MVMP score is the best proxy of trophoblast invasion-ischaemic injury-placental stress (Romero et al., 2018), and indeed it was strongly elevated in our early preeclampsia cases with or without HELLP syndrome, but not in late preeclampsia, also suggesting a similar placental pathology in early preeclampsia and HELLP syndrome (Varkonyi et al., 2011; Haram et al., 2017). When correlating histopathological signs of placental MVMP with perlecan immunoscores, we found a positive correlation in all cases of preeclampsia, but not in controls, suggesting a link between perlecan overexpression and problems with trophoblast invasion and placental perfusion in this syndrome. Moreover, perlecan immunoscore negatively correlated with placental weight among patients with preeclampsia; however, this was not the case in controls, reflecting that perlecan expression is also linked to restricted placental development due to decreased placental perfusion. Our data on the placental overexpression of perlecan in both preterm preeclampsia and miscarriages suggest that perlecan overexpression is related to the trophoblast invasion-ischaemic injury-placental stress pathological pathway found in both preterm preeclampsia and miscarriages. It would be interesting to test in vitro or in vivo in later studies how perlecan contributes to placental pathophysiology in these syndromes.

### Strengths and limitations of the study

The strengths of the study are: (1) Strict clinical definitions and homogenous patient groups; (2) standardized, quick placental sample collection during pregnancy terminations and C-sections; (3) standardized histopathological examination of the placentas based on international criteria; (4) protein expression profiling on first and third trimester placentas with tissue microarray and immunostaining followed by semiquantitative immunoscorings and statistical analysis; and (5) correlation of perlecan expression with clinico-pathological parameters of the placenta and the fetus.

A limitation of the study was the relatively modest number of cases in each third trimester group due to the strict clinical and histopathological inclusion criteria we used for patient enrollment. On the other hand, this was one of the most important strengths of our study. Another limitation was the use of third trimester early controls who had episode of preterm labor leading to preterm birth. These women had no clinical or histological signs of chorioamnionitis, no other medical complications, and delivered an AGA neonate, and thus were appropriate for gestational age matched controls. The expression changes of perlecan were studied using first and third trimester placentas. However, samples of second trimester could not be collected due to ethical reasons. Therefore, our results do not represent longitudinal pregnancy data, only reflect the beginning and the end of pregnancy. In addition, we focused on the investigation of perlecan expression in the syncytiotrophoblast due to the characteristics of our tissue collections. It would be interesting to compare perlecan expression in the extravillous trophoblast between various patient groups both in first and third trimesters using a different placental cohort.

## Conclusions

Our findings suggest that an increased placental perlecan expression may be associated with hypoxic ischaemic injury of the placenta in miscarriages and in early preeclampsia with or without HELLP syndrome.

## Supplemental Information

10.7717/peerj.6982/supp-1 Supplemental Information 1 Immunoscores and statistics of Figure 2. Raw data containing immunoscores and statistics of selected first and third trimester placentas.

10.7717/peerj.6982/supp-2 Supplemental Information 2 Immunoscores and statistics of Figure 3. Raw data containing immunoscores and statistics of first trimester placentas.

10.7717/peerj.6982/supp-3 Supplemental Information 3 Immunoscores and statistics of Figure 4. Raw data containing immunoscores and statistics of third trimester placentas.

10.7717/peerj.6982/supp-4 Supplemental Information 4 Glycan types and their compositions and structures in purified and placental MP4. Blue square/HexNAc: *N*-acetylglucosamine; green circle/Hex: mannose; yellow circle/Hex: galactose; red triangle/Fuc: fucose; purple diamond/NeuAc: sialic acid.

10.7717/peerj.6982/supp-5 Supplemental Information 5 The distribution of perlecan immunoscores in first trimester study groups. **A:** The number of placentas, representative cores and villi immunoscored in each first trimester study group are shown on the first panel. There was no villi with zero immunoscore. **B:** There was a larger proportion of villi with 3+ immunoscore in early pregnancy loss (EPL, 21%) than in first trimester controls (TOP, 11%).

10.7717/peerj.6982/supp-6 Supplemental Information 6 The distribution of perlecan immunoscores in third trimester study groups. **A:** The number of placentas, representative cores and villi immunoscored in each third trimester study group are shown on the first panel. There was no villi with zero immunoscore. **B:** There was a larger proportion of villi with 3+ immunoscore in early preeclampsia without HELLP syndrome (11%) than in third trimester early controls (1%).

10.7717/peerj.6982/supp-7 Supplemental Information 7 Negative control immunostaining. Negative control immunostaining was prepared without using primary antibody. Third trimester placenta, representative image, hematoxylin counterstain, 400x magnification.

## ABBREVIATIONS

BSA: bovine serum albumin
CID: collision induced dissociation
DAB: 3,3′-diaminobenzidine
DTT: 1,4-dithiothreitol
EPL: early pregnancy loss
FFPE: formalin-fixed paraffin-embedded
HPLC: high performance liquid chromatography
IAA: iodoacetamide
MP: membrane-associated placental protein
MS: mass spectrometry
MVMP: maternal vascular malperfusion
N_2_: nitrogen
NH_4_HCO_3_: ammonium bicarbonate
PE: preeclampsia
SDS-PAGE: sodium dodecyl sulfate poliacrilamyde gel electrophoresis
SE: standard error
TBST: tris based saline with 0.05% Tween-20
TFA: trifluoroacetic acid
TMA: tissue microarray
TOP: elective termination of pregnancy
Tris HCl: tris hydrochloride

## Additional Information and Declarations

### Data Availability

The following information was supplied regarding data availability: The raw data (immunoscores) are available in the Supplemental Files.
