# Supplementary figures and images for: Proteomic identification of membrane-associated placental protein 4 (MP4) as perlecan and characterization of its placental expression in normal and pathologic pregnancies
Source: PeerJ. 2019 Jun 20;7:e6982. doi: 10.7717/peerj.6982 (PMC6589330; doi:10.7717/peerj.6982)

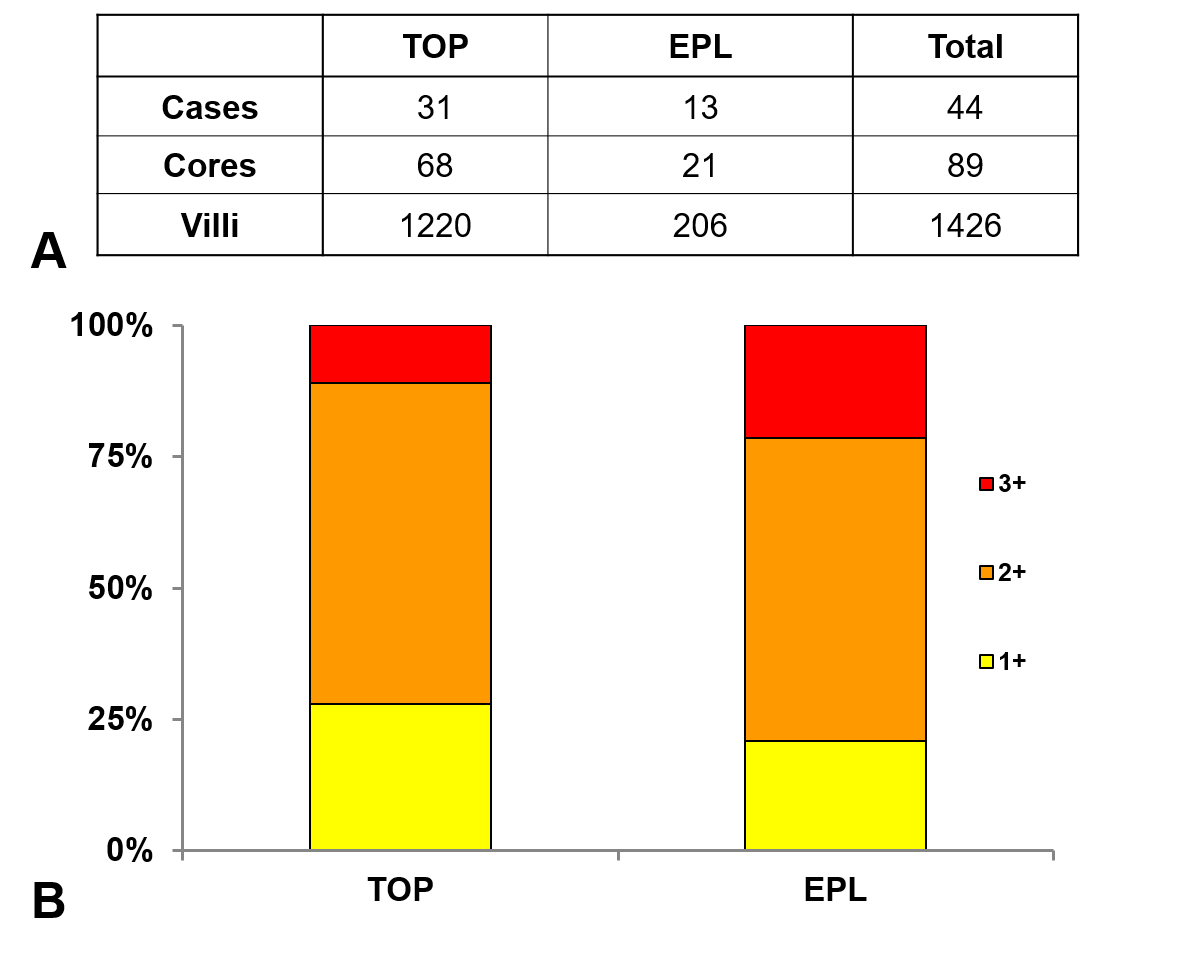

Supplement: Supplemental Information 5 — A: The number of placentas, representative cores and villi immunoscored in each first trimester study group are shown on the first panel. There was no villi with zero immunoscore. B: There was a larger proportion of villi with 3+ immunoscore in early pregnancy loss (EPL, 21%) than in first trimester controls (TOP, 11%). [file peerj-07-6982-s005.png]

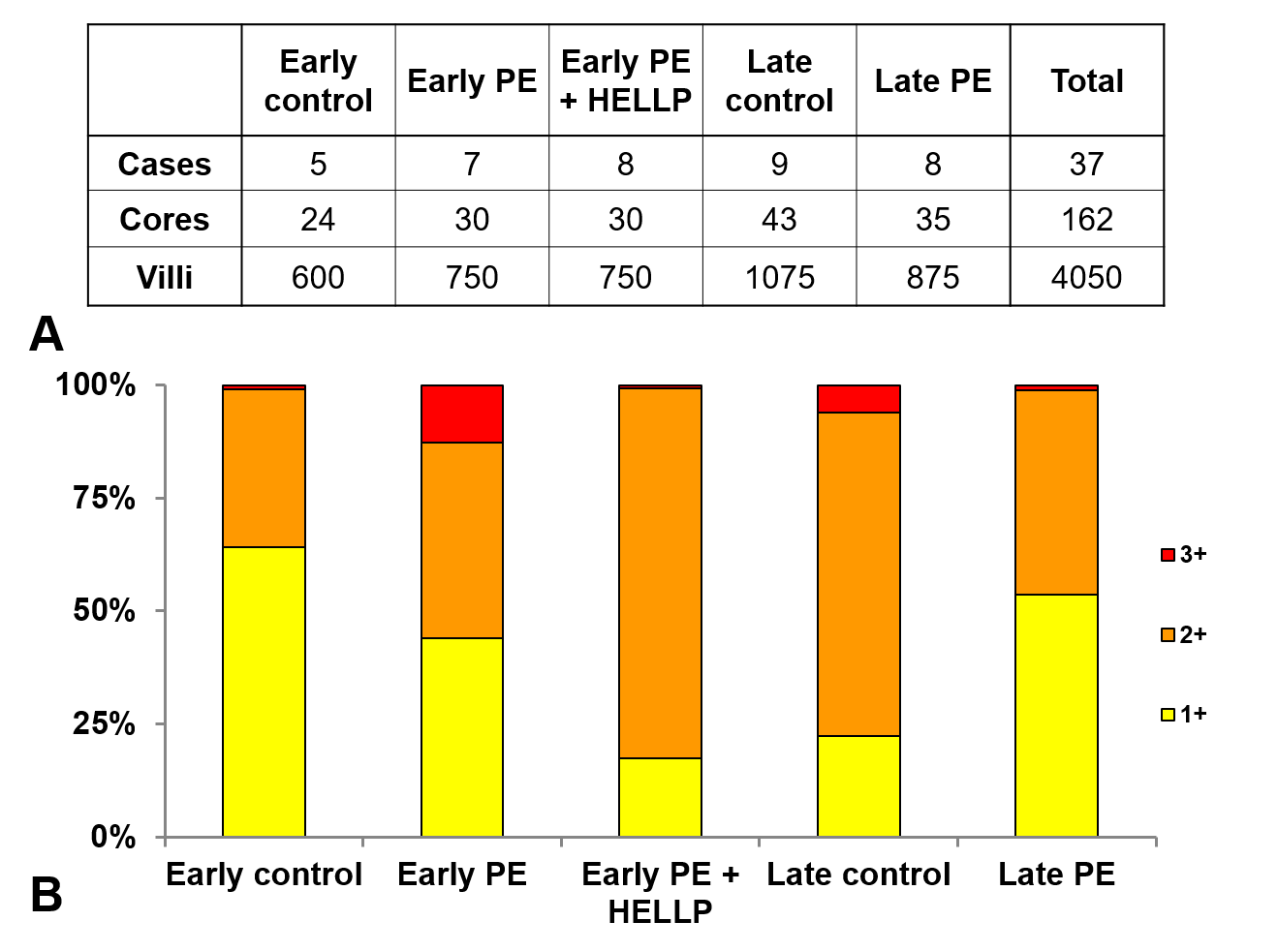

Supplement: Supplemental Information 6 — A: The number of placentas, representative cores and villi immunoscored in each third trimester study group are shown on the first panel. There was no villi with zero immunoscore. B: There was a larger proportion of villi with 3+ immunoscore in early preeclampsia without HELLP syndrome (11%) than in third trimester early controls (1%). [file peerj-07-6982-s006.png]

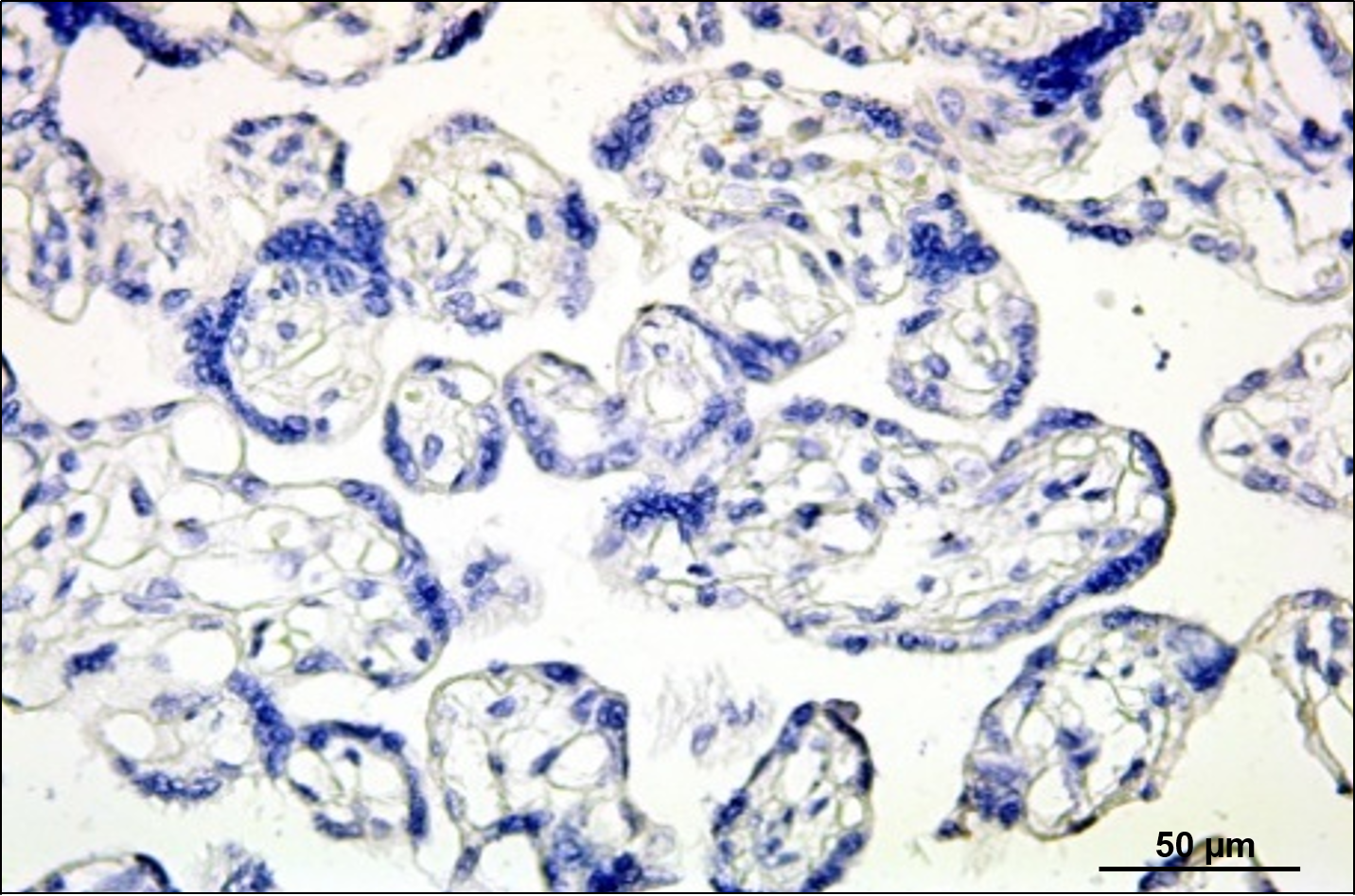

Supplement: Supplemental Information 7 — Negative control immunostaining was prepared without using primary antibody. Third trimester placenta, representative image, hematoxylin counterstain, 400x magnification. [file peerj-07-6982-s007.png]
